# Commentary on: High-Dose Neuromodulators: A Roundtable on Making Sense of the Data in Real-World Clinical Practice

**DOI:** 10.1093/asjof/ojab044

**Published:** 2021-11-13

**Authors:** Sachin M Shridharani

**Affiliations:** Washington University in St. Louis, School of Medicine, Division of Plastic and Reconstructive Surgery, St. Louis, MO, USA

Botulinum toxin type A (BoNTA) injection is the most frequently performed cosmetic procedure worldwide. Interest in optimizing outcomes, maximizing treatment duration, and improving efficacy has prompted the study of high-dose BoNTA treatment. “High-Dose Neuromodulators: A Roundtable Discussion on Making Sense of the Data in Real-World Clinical Practice” presents a roundtable discussion on the implications and findings of recent high-dose BoNTA studies for abobotulinum toxin A (ABO), incobotulinum toxin A (INCO), and onabotulinumtoxin A (ONA) (Video).^1^ Expanding on the findings derived from BoNTA pivotal trials is crucial to our understanding of dose on aesthetic outcomes, and I applaud the authors for providing an insightful examination and interpretation of the data on high-dose BoNTA treatment. Several key findings were presented in this article. Importantly, high-dose ABO data demonstrated that nearly all (≥89%) responding patients reported natural-looking results at all doses and timepoints (through 36 weeks). These findings were consistent with data from high-dose ONA studies. Accordingly, administering increased BoNTA doses in small volumes allows for natural outcomes and does not increase the potential for a “frozen” appearance. Regarding duration of effect, all high-dose studies demonstrated similar findings. Specifically, increased BoNTA dose correlated to incremental improvements in effect durability, regardless of the endpoint measure used. That said, while an increase in effect duration was seen with higher-dose treatments, the range was relatively narrow. For example, INCO data demonstrated the median duration of effect for 20, 50, and 75 units to be 177, 185, and 210 days, respectively. Notably, the change in duration of effect is not proportional to the corresponding change in dose, and the benefit of increased dose on outcome longevity largely plateaus at higher doses. Depending on individual patient goals, the incremental increase in duration of effect may not adequately justify the associated increase in treatment cost. Across all high-dose studies, treatment-emergent adverse events were not significantly higher among patients in higher-dose groups as compared with approved-dose groups. Overall, this article presents an informative discussion of recent studies on high-dose BoNTA and offers valuable insight from experts in our specialty on the important considerations and implications of high-dose treatment.
